# Defined by blood: lived experiences of menstrual health through the lifecourse among women in Zimbabwe – a qualitative study

**DOI:** 10.1136/bmjopen-2025-113914

**Published:** 2026-07-02

**Authors:** Mandikudza Tembo, Helen A. Weiss, Portia Nzombe, Chipo A. Nyamayaro, Fadzai Hove, Faith R. Kandiye, Ethel Dauya, Rashida A. Ferrand, Constance R. S. Mackworth-Young

**Affiliations:** 1 Biomedical Research and Training Institute, Harare, Zimbabwe; 2 Department of Global Health and Development, Faculty of Public Health and Policy, London School of Hygiene and Tropical Medicine, London, UK; 3 International Statistics and Epidemiology Group, London School of Hygiene and Tropical Medicine, London, UK; 4 Clinical Research Department, London School of Hygiene and Tropical Medicine, London, UK

**Keywords:** QUALITATIVE RESEARCH, PUBLIC HEALTH, Adolescent, Health Services Accessibility, Delivery of Health Care, Integrated, Dysmenorrhea

## Abstract

**Abstract:**

**Background:**

Menstrual health (MH) is vital to women’s overall health and well-being. Yet many women, especially in resource-limited settings, face challenges due to insufficient resources and support. Most MH research has focused on school-going adolescents while limited research has investigated MH experiences across the lifecourse. This study aims to understand menstrual experiences across women’s lifecourse in Zimbabwe, including MH-related pain and discomfort and women’s MH-related healthcare seeking behaviours.

**Methods:**

From February to April 2024, we conducted eight participatory workshops in Harare and Bulawayo, Zimbabwe with a total of 53 women aged over 18 years. Workshops aimed to explore lived experiences of MH and included participatory collage activities. We conducted thematic and visual analyses of the workshops and participant collages to explore participants’ MH perceptions and lived experiences.

**Results:**

Menstruation was seen as a central signifier of fertility and embodiment of womanhood. Women of all ages had limited access to education and support for menstrual-related issues (particularly menarche and menopause). Stigma and ill-informed healthcare providers were key barriers to menstrual-related healthcare and treatment. Women reported using alternative methods, including traditional herbs, while younger women, in particular, turned to online platforms to address menstrual-related issues.

**Conclusions:**

MH is central to women’s identity, health and well-being across the lifecourse. Persistent stigma, inadequate support and limited healthcare, especially during transitions such as menopause, highlight the need for inclusive policies and programming and adequately trained providers. Further research into digital and community-based MH approaches that empower women to navigate stigma and access care independently is also needed.

**STRENGTHS AND LIMITATIONS OF THIS STUDY**

- This study incorporates the experiences of adult women living in communities, expanding a knowledge base that has traditionally centred on adolescent girls in school.
- The study uses innovative participatory approaches to meaningfully engage with participants and carefully investigate menstrual health, an issue traditionally obscured by stigma and secrecy.
- The study investigates a broad spectrum of menstrual health-related experiences, rather than just focusing one specific aspect of menstruation such as menarche or menstrual stigma and taboo.
- Study findings may not be generalisable and may not reflect the lived experiences of those in rural settings as participants were only recruited from urban areas.
- The small number of workshops in the study may have limited the depth of our findings.

## Introduction

 Menstrual health (MH), defined as having access to the materials, facilities and support to manage menstruation with privacy and dignity, is integral to women’s overall well-being.^1^ It is important that MH-related challenges across the lifecourse are understood and addressed to mitigate gendered hygiene-related inequalities and related broader health, social and economic impacts.^1 2^ Improving MH will contribute towards achieving the Sustainable Development Goals (SDGs) by 2030, particularly SDG3 (Good Health and well-being), SDG4 (Quality Education) and SDG5 (Gender Equality).^3^ Current research shows that MH is integral not only to sexual reproductive health but to overall well-being.^4^,^6^ This highlights the need for (1) MH to be addressed across the lifecourse of women, not only in adolescence and (2) researchers, policymakers and providers to consider MH-related healthcare and support a fundamental component of physical and mental healthcare provision.^7 8^

**Figure 1:**
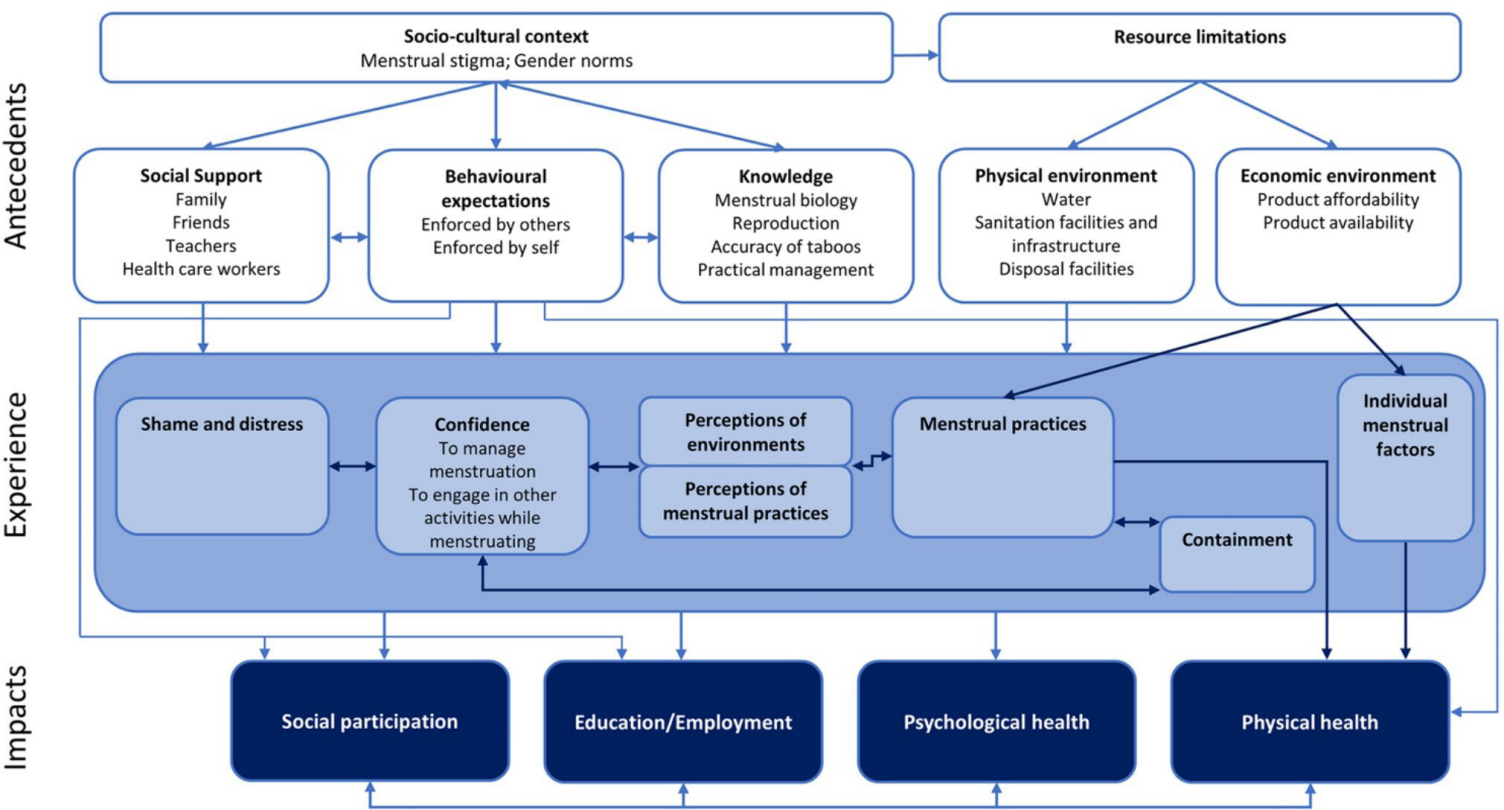
Integrated model of menstrual experience.^11^ Bolded headings capture themes, with subthemes presented below in unbolded text. Arrows depict directional and bidirectional relationships between themes.

MH is often compromised in resource-limited settings.^1 9 10^ An evidence-based integrated model of the distal (secondary) and proximal (primary) factors that shape menstrual experiences and affect well-being showed that key barriers to effective MH management include a lack of access to effective menstrual products, pain management, timely education and support, and water, sanitation and hygiene (WASH) facilities^11^ (figure 1). These challenges can lead to adverse outcomes including school drop-out, loss of income due to reduced participation at work and poor physical and psychosocial outcomes.^1012^,^17^ The evidence to date, however, focuses on school-aged girls and overlooks the experiences of women across their lifecourse, especially in older age. Also, few studies focus on other MH-related issues such as menopause and MH-related pain. There is an evidence gap on how the integrated model for menstrual experiences fits for adult women in low- and middle-income countries (LMICs), looking at MH experiences and challenges across the lifecourse, and/or at other critical aspects of MH such as pain management and discomfort.

In Zimbabwe, as in many other settings, MH needs are inadequately addressed.^18^ Many women face challenges in accessing care for MH-related issues, clean water or private spaces to attend to their needs in schools, homes and workplaces and use unhygienic and uncomfortable menstrual materials that may negatively affect their health, education and well-being.^13 19^ In 2019, the Zimbabwe Multiple Cluster Indicator Survey reported 16.3% of women aged 15–49 years did not participate in social activities, school or work due to menstruation at least once in the last 12 months.^20^ Another mixed-methods study of MH among 27 725 young women aged 16–24 years in Zimbabwe revealed similar findings with most reporting experiences of MH-related pain and other related disorders such as heavy bleeding, inadequate information and support on pain management and missed school or work due to MH-related issues.^21^ While the existing literature on MH in Zimbabwe gives some insight into the challenges girls and women face in managing MH, this work is limited to a few studies that mostly focused on younger women.

The aim of this study was to understand the menstrual experiences across the lifecourse of women in Zimbabwe, including investigating managing menstruation, MH-related pain and discomfort, and the barriers and facilitators to seeking MH-related healthcare and support.

## Methods

### Study design and setting

This qualitative study was conducted between February and April 2024 in urban settings in two provinces in Zimbabwe (Harare and Bulawayo). These provinces were selected as they are the largest urban areas and their populations represent the two largest ethnic groups in the country, Shona and Ndebele. Zimbabwe is a landlocked country with a population of 15 178 979, of whom 52.3% are female.^22 23^

In Zimbabwe, the socio-economic limitations, fragile health systems, poor WASH facilities and deep-rooted gendered taboos severely undermine women’s ability to manage MH with dignity and safety, compromising their health, education and broader socio-economic opportunities. In Zimbabwe, poverty is pervasive with 42% of the population living on less than US$1.90 per day.^24^ A pack of disposable pads costs US$0.50–2.00 and a packet of five reusable pads costs US$4.00–10.00, with the cost increasing with the quality of the product. Most Zimbabweans cannot afford private healthcare and public healthcare access is limited by systemic challenges, including under-resourced facilities, geographical disparities in service availability and associated high transport costs, unaffordable user fees and limited skilled healthcare personnel and essential medical supplies, particularly in maternal and reproductive health.^25 26^ In terms of water and sanitation infrastructures, the majority of public schools and facilities lack private, lockable toilets, water, soap or safe disposal facilities like incinerators or sanitary bins.^27^ Activities and education are often gendered, including widespread exclusion of boys and men from MH education and information.

### Study participants

Working with community-based organisations and community healthcare workers who regularly interface with girls and women in the study communities, we aimed to recruit 30 women per province. All women aged 18 years and older with capacity to provide informed consent were eligible to participate. We recruited women through clinic and community outreach. Trained female qualitative research assistants (RAs) (PN, CAN, FH, FRK) contacted participants in person or via phone call with information about the study and invited them to enrol. To ensure a range of MH experiences were represented, we also actively recruited some women with bleeding disorders, perimenopausal women and women who have sought formal or informal care for MH-related issues in the past. This targeted recruitment allowed for a more comprehensive understanding of the MH experiences and needs of women in the communities. Written informed consent was obtained before the workshops were initiated. The participants were purposively disaggregated by age in the workshops to represent MH experiences of older versus younger women in the communities.

We recruited 60 women; however, 7 failed to show up on the day scheduled for the workshop due to transport issues (3), sickness (1) or other commitments (3). The criterion for this study was women across different life stages regarding the menstrual cycle. Participant age categorisation was informed by Phipps *et al*^28^ study that suggests postmenopausal cut-off age of 55 years. We therefore used a categorisation of younger (reproductive-age) women aged 18–35 years and older women, including both midlife (perimenopausal) women aged 36–55 years and postmenopausal women aged 55+ years.

We conducted eight participatory workshops with 53 women aged 18 years or older, with four participatory workshops in each province (two with younger women aged 18–35 years and two with older women aged >36 years and older) (table 1). All participants came from low-income households situated in high-density areas. Most households in these areas have limited access to WASH facilities, with most accessing water via a public well and some having access to a toilet and washing facilities in the household and others sharing access to a toilet and washing facilities outside the household.

**Table 1:** Participatory workshop participant demographics

		Female participants (n=53)	
			n
Age	Younger	18–35 years	32
	Older	36–55 years	13
		56–101 years	8
Province		Harare	26
		Bulawayo	27

### Data collection: participatory workshops

Workshops (each with 12–15 participants) were facilitated by an experienced social scientist (MT) and four trained qualitative RAs (PN, CAN, FH, FRK). They were conducted in Shona and English in Harare and in Ndebele and English in Bulawayo (as preferred by the participants), lasting 120–180 min, with a refreshment break. We developed semistructured topic guides for the workshops, informed by findings from a previous study with young women in the same setting (17, 28) and research team discussions (with HAW and CRSM-Y and led by MT). Each workshop included group member introductions and guided small (two to three participants) and large (all participants together) group discussions. The discussion questions and activities were used to explore participants’ perspectives on and lived experiences of managing menstruation and seeking healthcare for MH-related issues. The workshops also included a participatory collage-making activity.^29^ Created individually in the shared workshop space, participants used magazine cut-outs, markers and menstrual products to showcase their lived experiences of managing their MH. Participants then described the collages to the larger group, including the particular meanings of the different collage elements. The activity encouraged participants to narrate stories about their MH experiences within the wider context of their everyday lives and activities in a non-intimidating creative way while interacting with other participants.

Workshops were audio-recorded and recorded through detailed note-taking which included features of the workshop setting, non-verbal interactions between participants and facilitators’ reflections on workshop activities as methods for understanding the experiences, perceptions and opinions of the participants. Original collages were collected and archived for analysis. Pseudonyms were used throughout for anonymity. Verbatim quotes were captured.

### Data analysis

Data analyses were guided by Braun and Clarke’s approach to thematic analysis.^30^ During analysis, we focused on understanding participants’ experiences of MH, managing menstruation and seeking MH-related healthcare and support. Data included in analysis were transcriptions of workshop audio recordings, detailed workshop notes and artwork from the collage-making activity. From familiarisation of the data, codes and themes were identified through qualitative analytical discussions between CRSM-Y and MT.^31^ Data were manually coded using Microsoft Excel. Example codes included lack of preparedness for menarche, limited access to MH-related education and support and the importance of fertility, cleanliness and menstruation in performing and embodying womanhood. Collages were analysed visually and thematically by the team who together iteratively discussed themes^29 32^ through laying out the collages grouped according to shared themes. Notes were taken, and themes were discussed iteratively by the team, comparing with the themes that emerged from the other workshop activities. Analytical memos with quotes and visuals were written thematically to organise and analyse emerging findings from all the written and visual data and formed the basis for iterative analytical discussions and the results presented below.^33^

### Patient and public involvement

This study included public involvement in the conduct of the research. Briefly, we used participatory workshops with women aged 18 years and older to investigate the lived experiences of MH across the lifecourse of women in Zimbabwe.

### Researcher characteristics and reflexivity

The research team consisted of nine women who identify themselves as cis-gendered. Five are Zimbabwean and local to the study area (MT, PN, CAN, FH, FRK and ED), two British born (HAW and CRSM-Y) and one is from Pakistan (RAF). HAW has visited Zimbabwe multiple times and has done extensive MH-related research in the region. CRSM-Y relocated to Zimbabwe in 2022 and RAF has lived and worked in Zimbabwe for over 20 years. The entire team has research experience working with sexual and reproductive health and rights, including MH. MT is an MH specialist and postdoctoral researcher. PN, CAN, FH and FRK are RAs with years of field experience. ED is a public health specialist and field director. HAW and RAF are senior researchers and professors at the London School of Hygiene. CRSM-Y is a public social scientist and an assistant professor at the London School of Hygiene and Tropical Medicine. The varying professional disciplines and perspectives made it possible to view and interpret the data from different angles which enriched the overall data analysis and understanding. However, the researchers’ characteristics, prior knowledge and personal experiences and biases may also have informed how they interpreted the data and discussed the findings.

## Results

### Sociocultural constructions of menstruation

#### Menstruation as a marker of fertility and womanhood

Almost all participants, regardless of age, felt menstruation marked the beginning of ‘womanhood’ as it signified that one’s body was biologically ready to conceive a child. Menstruation signified fertility and was considered a key aspect of the transition into womanhood and the potential for transitioning to motherhood, as strongly linked to womanhood:

When you get your period, it means you are growing up. It means you can now get pregnant and have children… – early 20s, Harare

Due to this linkage between menstruation and fertility, participants also discussed how, when they started menstruating, they were told to stop ‘playing with boys’ as this could lead to unwanted consequences such as pregnancy:

When I started my period, I was told not to hand shake any boys because I was told that if I do so I will bleed more, so I would often run away from them because I didn’t want that. – mid 40s, Bulawayo

The perception that menstruation was a key indicator of fertility meant that, despite the pain, discomfort and burdensome practices associated with it, most participants, regardless of age, described menstruation as a ‘gift’ and an important part of their lives.

Despite all this, I am glad that I have my five children as a result of my periods… – mid 30s, Bulawayo

The perception of menstruation as a gift was also noted as older women reflected on menopause. While some celebrated the end of menstruation and the end of the burden of its management, most mourned the loss of an important part of their identity as women:

Periods are precious. When I stopped my period, I was shocked… I was no longer a young woman. At least I had my children, but it was still a shock. It was a sign that now I was really old, a real gogo (grandmother or old woman) – early 40s, Bulawayo

#### Menstruation as a secret

**Figure 2:**
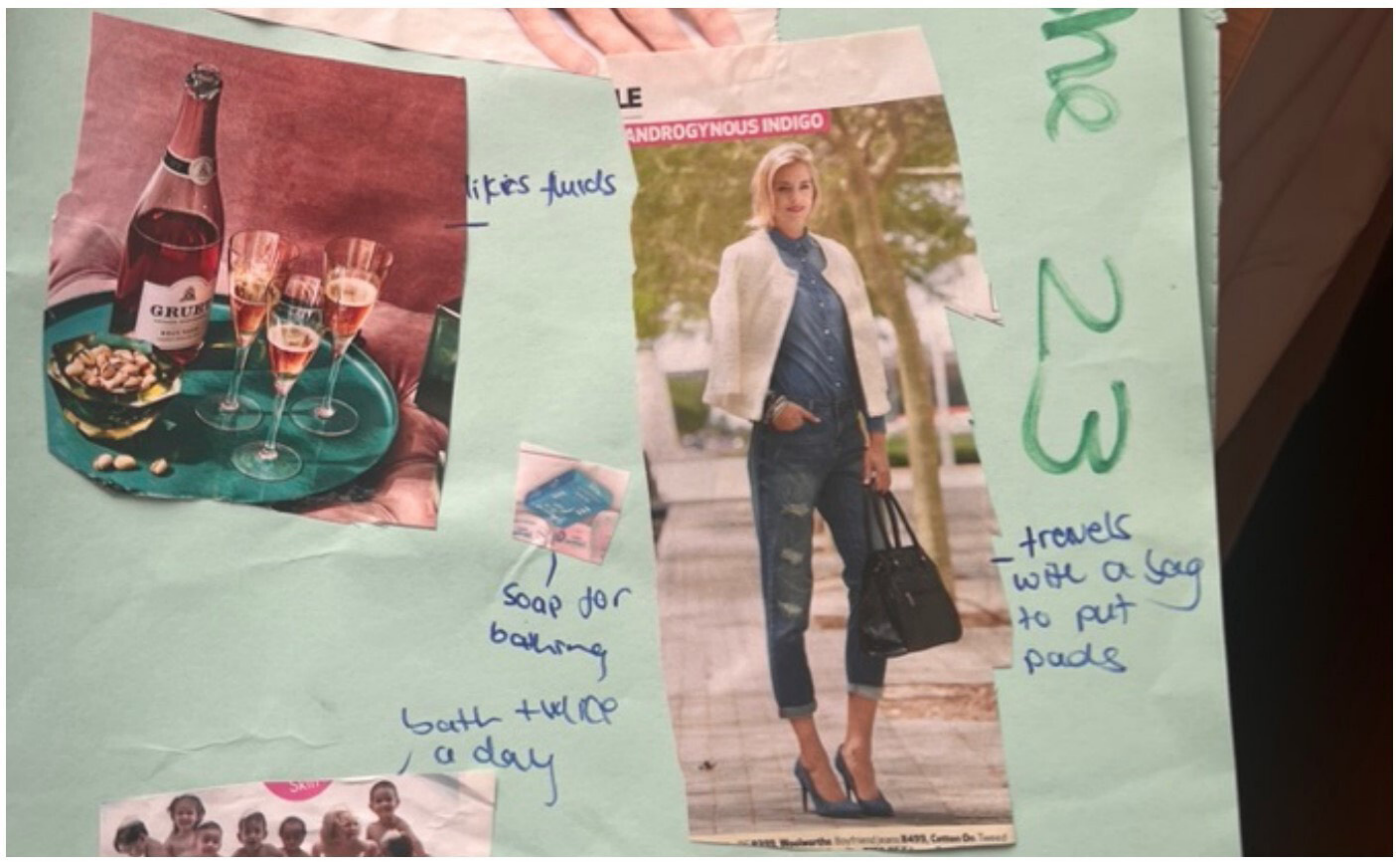
Collage by woman in early 20s, showing a bag to hide pads in.

While participants openly spoke about menstruation in the workshops, they noted that, in general, menstruation was considered a personal and private matter. Many participants described being told to keep their periods a secret. They reflected on how practices such as frequent bathing or “travelling with a bag to [hide] menstrual products” (see figure 2) were done to make sure no one could tell if one was menstruating:

You need to be extra hygienic; you need more toiletries than you already have. No one must know that you are on that time of the month. You need to be clean, and you need to bath more and to smell good – early 20s, Harare

Participants also mentioned how the secrecy around menstruation negatively informed their experiences of menarche as some had never had discussions around menstruation with their parents or peers. They therefore felt that there was no one they could tell or seek support from when they started menstruating:

Growing up it was a taboo to even mention that you on your period… Even if you had a period pain, you had to soldier on or even lie that you have a headache because it’s something that was just not up for discussion… – mid 50s, Bulawayo

Older participants added that these negative experiences at menarche continued to negatively inform their perceptions and experiences around menstruation and MH-related issues throughout their lives. Some participants expressed feeling relieved of ‘burden’ once in menopause.

Periods have always felt like a burden. It is continuous work and pain. I was actually happy when they stopped. – mid 50s Harare

#### ‘My blood is dirty’: negative perceptions around menstrual blood

All participants discussed being told that menstrual blood was ‘dirty’ or ‘dangerous’. If they shared that they were menstruating, their participation in social, economic and religious activities were limited due to beliefs of menstruation making one unclean:

I grew up in the rural areas and I was told not to cook for my grandfather while on my period. I was also told not milk my father’s cows and goats. I was also told not to eat milk while on my periods because it would result in my grandfather’s cattle and goats failing to produce any milk. – mid 20s, Bulawayo

For me once one is on their period we are not allowed to cook or do anything for our husbands. I am considered to be unclean so even washing for him or sharing the same bed is not allowed. During this time, there is no bond between husband and wife. – late 30s, Bulawayo

**Figure 3:**
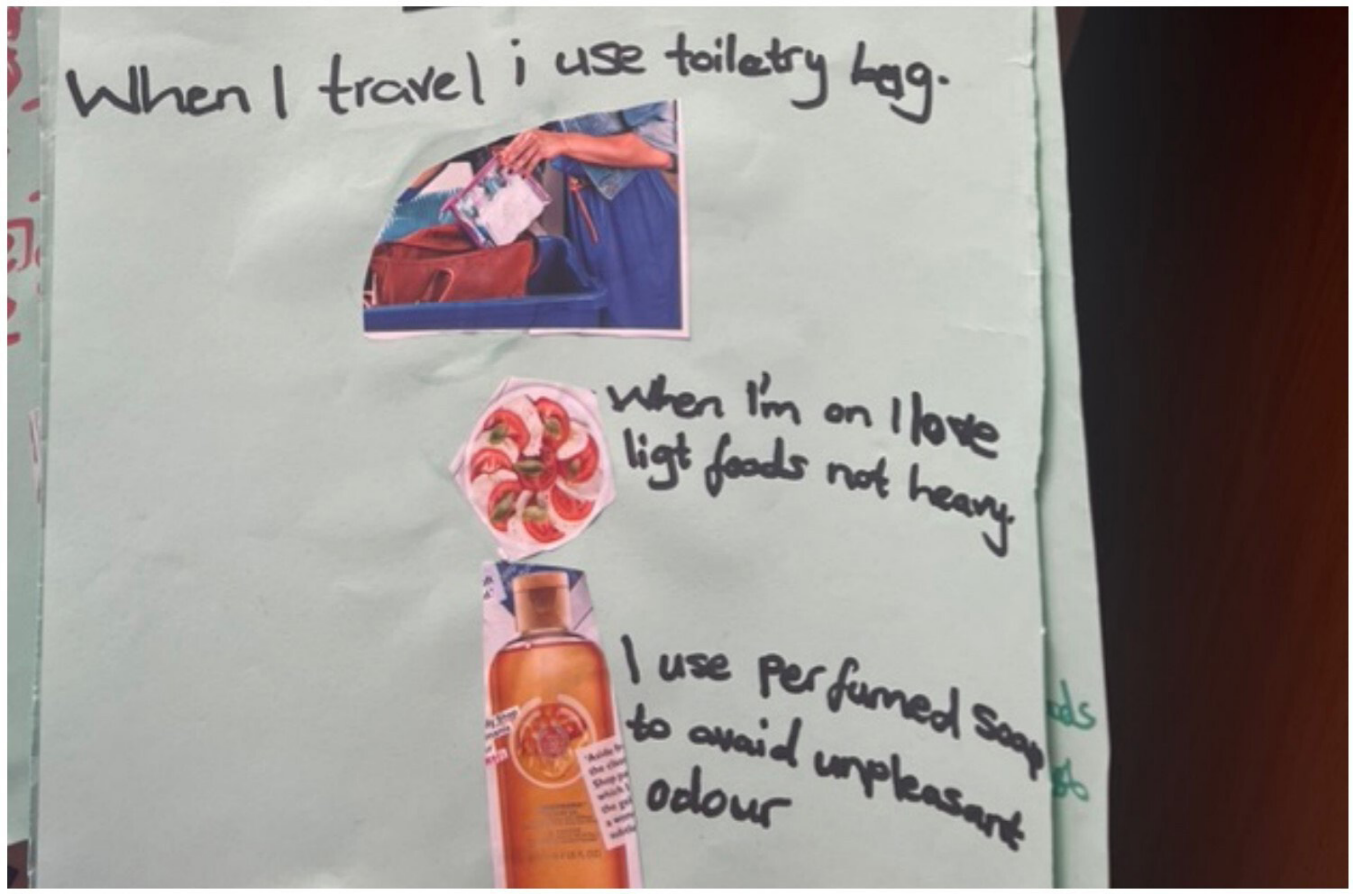
Collage by woman in early 20s showing use of perfumed soap to avoid unpleasant odour during menstruation.

**Figure 4:**
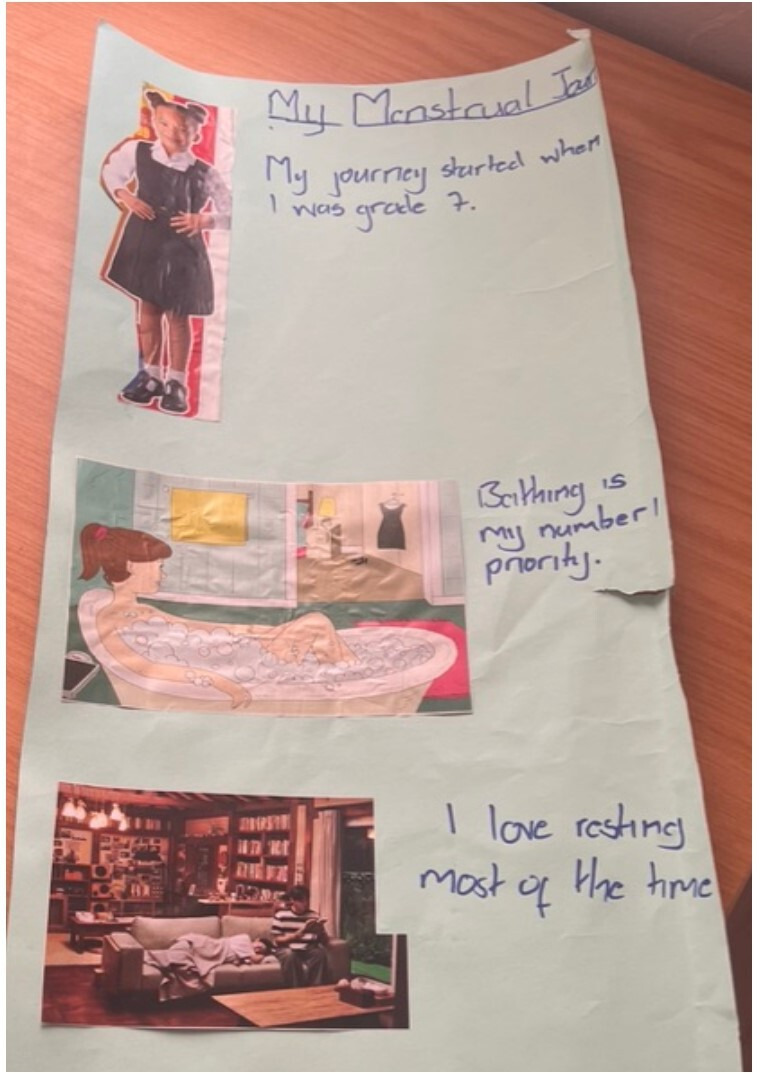
Collage by woman in mid 20s describing bathing practices during menstruation.

This perception of menstrual blood being unclean led participants to use more soap and scented products once they started menstruating (see figure 3). Participants also described being instructed to bathe more often when they had started menstruating (see figure 4):

When you are on your period, it is important to bath well so you don’t smell. My mother told me that… So, at the beginning I was bathing three or four times a day (laughing) – early 20s, Harare

Such practices of cleanliness to conceal menstruation were considered a lifelong responsibility and burden.

While most older participants agreed with the belief that menstrual blood was unclean and felt such limitations in social, economic and religious activities were reasonable, most younger women felt these beliefs were harmful and untrue, and should be revised:

Men and women should be taught that there is nothing wrong and dirty about the period. That way even those who have questions about their period will not feel ashamed to ask if there are pressing issues – early 30s, Bulawayo

Here, many younger women noted that exposure to MH-related information from online resources, period-tracking applications and MH advocates changed their perceptions around menstrual blood and menstruation to be more positive.

### Limited access and agency in managing menstrual health

When asked about their experiences of managing menstruation, many participants reported feeling isolated and alone. Almost all the participants spoke about feeling unprepared for menarche and then managing MH-related issues throughout their lives.

#### Unprepared and uninformed: first experiences of menstruation

Many participants recalled that their first few months of their periods were particularly difficult as they did not know what was happening to them or what to do to manage the blood flow and/or pain:

I started my period when I was in high school. My mother was not that open with me, so when I started, I didn’t tell her because I thought something was wrong with me. I ended up using pieces of cloths as pads because I couldn’t ask my mother to buy me pads – early 60s, Bulawayo

Some younger women noted that they had access to some information about menstruation and MH-related information from school, discussions with peers and/or online sources. However, almost all older women reported feeling uncomfortable talking about menarche with peers and having had little knowledge of where to find MH-related information or support when needed. Some also reported being unaware and surprised about other types of MH-related issues such as postpartum bleeding or heavy and/or irregular bleeding due to family planning methods such as the hormonal contraceptive implant or the copper intrauterine device.

Particularly for older women, this lack of information or support has resulted in lasting negative feelings around menstruation such as shame and disgust which led to poor menstrual experiences. Here, their menarcheal experiences established the mood for their menstrual experiences across the life-course. A negative effect often comprising pain, solitude and anxieties linked to the need to always keep one’s MH-related issues a secret. Without the experience of talking about MH throughout their lives, many older women described not having the language or confidence to address MH-related issues linked to discomfort or pain and so they often suffered in silence either assuming their experiences were normal or too scared or ashamed to seek help.

#### Limited choice of MH products

**Figure 5:**
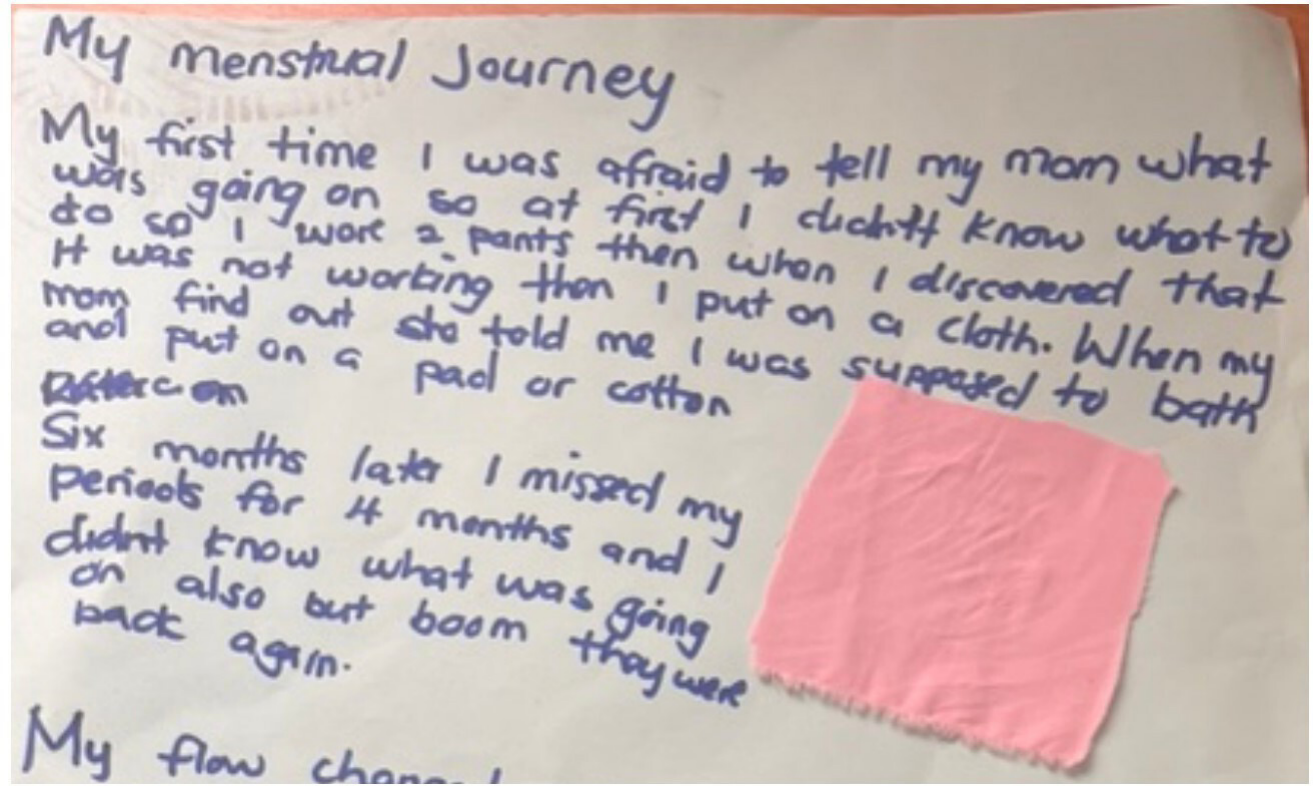
Collage by woman in early 40s describing menarche and showing cloth material used to manage menstruation.

In describing their menarcheal experiences, many participants reported using tissue or cloth for their first period as they were unprepared and afraid to seek advice on what to do and what to use (see figure 5). Feeling unprepared and having a lack of options for advice was universal among older women, while some younger women noted being able to ask a friend for assistance.

Most participants reported using cloth, reusable pads or disposable pads. While a few participants spoke about preference guiding their product choice, most used what was available at home, largely cloth, as they found most menstrual products too expensive:

Pads are very expensive… I use them when I can afford otherwise I use reusable pads that I make myself – early 30s, Bulawayo

Most older women reported feeling more comfortable with cotton or cloth even though they can now afford pads because this is what they are used to:

Where I came from, I don’t recall ever seeing pads or cotton wool. I remember at one time my father came for holiday and I stole a pair of socks so that I could use as pads because the type was very thick. Until today, I still use pieces of cloths because of my flow. I have tried using pads, but they have never worked for me – early 40s, Bulawayo

### Navigating MH-related issues: pain, care-seeking and MH technology applications

#### Pain, heavy bleeding and fertility concerns

Many participants reported having experienced menstrual-related pain and heavy bleeding, and associated menarche and menstruation as something to suffer through.

Periods is a time to suffer, we experience cramps, and we will not be comfortable to hang out with others, you just want to isolate yourself to meditate or something… – late adolescence, Harare

When I started my period, I was 15 and I had the worst experience because I had severe period pain and a heavy flow that went on for two weeks… Even if I take any painstop [medication] the pain doesn’t go away – early 40s, Bulawayo

Some reported that they had missed school and/or work due to extreme pain:

I have a severe period pain and I always want to sleep and not do anything. Sometimes I even skip going to school because of that and if it starts while I am at school, I sometimes ask for a pass to go back home. – early 20s, Bulawayo

Other MH-related issues mentioned by a few, mostly older participants, included fibroids, endometriosis and menopause. Participants reported knowing very little about these conditions and only being made aware of them when they visited a healthcare facility for symptomatic care.

When younger women were asked about issues such as menopause, most had limited knowledge about these issues and felt that they were *“*too young for such issues for older women*,”* (early 20s, Harare).

#### Management through healthcare: a last resort

Most participants noted that they had not sought healthcare services to address MH-related issues as they found healthcare services too expensive, and healthcare providers ill-informed or dismissive:

People go to the doctor after having tried free solutions first. – early 30s, Harare

For me I had issues with my period, but I never got comprehensive assistance on what I wanted. I had questions on why I had a period pain or why I had irregular bleeding, but I was just told that its normal to experience that during my period. I feel like the nurses are not well informed on these issues. – mid 50s, Bulawayo

Participants also noted that neither they nor their family members considered MH-related issues such as pain, heavy bleeding or irregular bleeding a health priority that necessitated medical attention:

Despite having a period pain, I have never gone to the clinic for that, growing up I was told that it’s just normal, so I didn’t see the need to go to the clinic. – mid 30s, Bulawayo

If help or support was needed, participants reported seeking out a trusted friend or older woman, and saw formal healthcare as a last resort:

It all depends on the matter, some of the things we just talk as friends but for matters that are complicated, we go to the hospital. – early 60s, Bulawayo

The notable exception to this reluctance to seek healthcare for MH-related issues was when they related to fertility, which was considered something ‘serious’:

I was trying to get pregnant but nothing was happening. I was bleeding here and there, heavy flow but that was normal for me. I just had to go to the hospital because I did not understand why I was not having a baby… I had to go for scans and everything. It was very expensive… – mid 50s, Harare

Overall, seeking healthcare management for MH-related issues was a last resort for most participants regardless of age.

#### Management through traditional medicine

Other management options that participants shared included the use of traditional herbs and practices and online resources. Both older and younger women mentioned using herbal concoctions to treat MH-related pain and heavy bleeding:

I don’t know the name of the tree… I think it’s ‘chinaberry barks’…they are bitter, but I drank them and the pain was better. – early 20s, Harare

For my period pain I have tried managing it by drinking ginger tea… The pain goes away for a short period of time then I must do it again – mid 30s, Bulawayo

Some participants also mentioned engaging in other practices to shorten and lighten menstrual flow:

I used to bleed for many many days and my grandmother told to sit on an anthill during my period… Don’t laugh… It worked for me. Now I only bleed for three days – early 20s, Harare

#### Management through use of technology

In addition to speaking to their peers and/or elders and using alternative methods such as traditional medicine, many younger women also reported using period-tracking applications (apps) and online platforms such as TikTok and Google.com to seek MH-related guidance and support:

For issues that deal with my period… I read about such matters on social media, like someone might post their problem asking for help on a certain issue. Answers that are given by other people are some of the answers that I apply in my own situations… – mid 30s, Bulawayo

Women also reported using MH-focused apps such as Flo and Clue to monitor period dates, symptoms, moods and behaviours, all of which enhanced their MH perceptions and practices:

If you subscribe to ‘Flo’, you can log your symptoms and there are doctors who can help you and you get advice on the application. – late 20s, Harare

Some younger women noted that apps and online platforms have become so popular that “[they] are causing people not go to the doctors at all,” (late 20s, Harare).

## Discussion

This study reveals the key role that MH plays in defining ‘womanhood’, the influence MH has on women’s everyday lives and engagement in activities, and the limited information, products and healthcare options for women in Zimbabwe. Our findings both support and add to the integrated model of menstrual experiences generated by the 2019 systematic review.^11^ We add a temporal lens to the model, elaborating on proximal and distal antecedents of menstrual experiences through to the impacts of this at different ages and exploring the similarities and differences of lived MH experiences across generations of women. By including the narratives from older women, our research highlights (1) the lifelong negative impact of persistent stigma and taboo around menstruation on MH experiences and management and (2) the lack of adequate support and healthcare for other MH-related issues, particularly menopause. The study also highlights the subsequent rise in the use of alternative methods and resources to manage MH-related issues, particularly MH-focused technology and media platforms. While research in this area has received increased attention, further research is needed to better understand their effectiveness, inclusivity, safety and overall impact on users^34 35^

Our findings highlight how negative menstrual experiences early in life can lead to internalised stigma and can have long-lasting adverse impacts on women’s overall well-being later on in life. Feelings of shame keep Zimbabwean women, particularly those from older generations, from seeking MH-related healthcare or support leaving them to suffer in silence.^36 37^ This study highlights how stigma hinders important discussions around MH and informs negative menstrual experiences. Critically, our study demonstrates that a lack of timely MH-related information and support was not isolated to their young lives, but a ubiquitous circumstance across the life course. MH stigma, a social construct, drives a system of processes to discredit, disempower and devalue those who menstruate.^38^,^40^ From early on, young women learn that menstruation is a dirty secret that should be concealed, leading to silence and shame. This stigma is then reinforced through interpersonal interactions and by institutions (schools, the media and the healthcare system), particularly in instances of failure to conceal MH through leaking, smelling or showing discomfort or pain.

Almost all women entered menarche with little to no knowledge of what menstruation was or how to manage it. Many recalled feelings of shock, surprise or horror at menarche and this negatively informed how they experienced and managed MH-related issues throughout their lives. These findings echo those in other studies looking at menstrual experiences among women in high-income countries, including Brantelid *et al*^8^ and Barrington *et al*.^7^

Our findings not only reveal barriers to healthcare seeking behaviour but also gaps within healthcare provision. Of particular concern is the inadequate knowledge and support regarding menopause and its management. This supports evidence from a scoping review of menopause in LMICs,^41^ highlighting the low prioritisation of MH-related issues among older women and the lack of perimenopause and menopause resources and support available to them in LMICs such as Zimbabwe.^42 43^

Our study highlights potential alternatives to manage MH-related issues. Specifically, the use of herbal remedies and/or the use of online MH communities/support groups and applications. Regarding the use of traditional and/or herbal medicines to treat MH-related issues, a recent study showed the practice is present in communities around the world with a total of 571 traditional and/or indigenous medicines being commonly used to treat conditions such as heavy bleeding and moderate to extreme pain.^44^ However, the efficacy and safety of many of these medicines/methods is limited and more research is needed before it can be considered and promoted as appropriate care for MH-related issues.

Another alternative to healthcare management is technology; online technology is increasingly being used to address MH, offering accessible and personalised resources, especially by young people. Digital platforms, including apps, websites and social media, are providing educational content, period tracking and a sense of community for managing MH.^45^ In our study there was a clear difference in MH knowledge and perceptions by age, with younger women having better knowledge and more positive perceptions around menstruation due to the use of menstrual cycle tracking applications and period positive rhetoric on social media. Another qualitative study looking at use of online information for MH learning among adolescent girls in Indonesia had similar findings.^45^ While more research is needed to evaluate effectiveness and safety around the use of technology to address MH needs, current evidence shows overall positive results.^46^

We also reveal the normalisation of MH-related pain. Women described menstruation as a natural bodily process that came with discomfort and pain. MH-related pain was expected and considered normal at both an individual level and institutional level as participants reported healthcare providers being dismissive and uninformed when it came to addressing their pain management. Similar findings have been reported globally.^7 11^

A strength of our study is the inclusion of adult women in communities, thus contributing important data to a knowledge base that has historically focused on adolescent girls and girls in school. We also asked about broad MH-related experiences, rather than focusing on specific aspects of menstruation such as MH symptoms, menarche or MH stigma and taboo. The study also faced some limitations. First, participants were only recruited from urban areas and thus we did not capture the lived experiences of those in rural settings. Second, the qualitative data may be subject to recall bias where participants were asked to narrate and discuss menstrual experiences across years, and for some decades, of their lives. Thirdly, the small number of workshops in the study may have limited the depth of our findings. Lastly, the public and social format of the participatory workshops may have constrained disclosure, especially around particularly sensitive or private experiences or opinions around MH. In future research, this limitation could be mitigated by including follow-up in-depth interviews to further explore sensitive experiences in a more private setting.

### Recommendations and conclusion

This study shows the central role of MH in lifelong experiences of women in Zimbabwe. Menstruation is viewed as a natural process, sign of good reproductive health and the beginning of womanhood. Almost all women had negative MH-related experiences during their lives, particularly at, but not restricted to, menarche and menopause. Challenges included longstanding negative perceptions of menstrual blood as ‘dirty’ and MH stigma, a lack of access to timely MH education and support, and barriers to MH-related healthcare provision, particularly for pain or MH-related issues in older age such as menopause.

Given the impact of MH on women’s overall well-being, implementors and policymakers should be encouraged to destigmatise and normalise MH by (1) increasing their commitment to timely, stigma-free and comprehensive MH-related healthcare provision, (2) diversifying access to MH sources of information and support by investing in innovative research and interventions such as community-based and online MH resources, and (3) prioritising other MH-related issues across the life course such as menopause. Furthermore, to support the development of evidence-based policies and practices, additional research is needed on digital and community-based MH approaches that empower women to navigate MH-related stigma and access care autonomously.

## Data Availability

Data are available upon reasonable request.

## References

[1] Hennegan J, Winkler IT, Bobel C (2021). Menstrual health: a definition for policy, practice, and research. Sex Reprod Health Matters.

[2] Cislaghi B, Heise L (2020). Gender norms and social norms: differences, similarities and why they matter in prevention science. Sociol Health Illn.

[3] United Nations (2015). Transforming our world: the 2030 agenda for sustainable developmen.

[4] Wilson LC, Rademacher KH, Rosenbaum J (2021). Seeking synergies: understanding the evidence that links menstrual health and sexual and reproductive health and rights. Sex Reprod Health Matters.

[5] Hoppes E, Rademacher KH, Wilson L (2023). Strengthening Integrated Approaches for Family Planning and Menstrual Health. Glob Health Sci Pract.

[6] Tembo M, Renju J, Weiss HA (2022). Integration of a menstrual health intervention in a community-based sexual and reproductive health service for young people in Zimbabwe: a qualitative acceptability study. BMC Health Serv Res.

[7] Barrington DJ, Robinson HJ, Wilson E (2021). Experiences of menstruation in high income countries: A systematic review, qualitative evidence synthesis and comparison to low- and middle-income countries. PLoS One.

[8] Brantelid IE, Nilvér H, Alehagen S (2014). Menstruation during a lifespan: A qualitative study of women’s experiences. Health Care Women Int.

[9] Plesons M, Patkar A, Babb J (2021). The state of adolescent menstrual health in low- and middle-income countries and suggestions for future action and research. Reprod Health.

[10] Shannon AK, Melendez-Torres GJ, Hennegan J (2021). How do women and girls experience menstrual health interventions in low- and middle-income countries? Insights from a systematic review and qualitative metasynthesis. Cult Health Sex.

[11] Hennegan J, Shannon AK, Rubli J (2019). Women’s and girls’ experiences of menstruation in low- and middle-income countries: A systematic review and qualitative metasynthesis. PLoS Med.

[12] Sivakami M, Maria van Eijk A, Thakur H (2019). Effect of menstruation on girls and their schooling, and facilitators of menstrual hygiene management in schools: surveys in government schools in three states in India, 2015. J Glob Health.

[13] Ssewanyana D, Bitanihirwe BKY (2019). Menstrual hygiene management among adolescent girls in sub-Saharan Africa. Glob Health Promot.

[14] Torondel B, Sinha S, Mohanty JR (2018). Association between unhygienic menstrual management practices and prevalence of lower reproductive tract infections: a hospital-based cross-sectional study in Odisha, India. BMC Infect Dis.

[15] Phillips-Howard PA, Otieno G, Burmen B (2015). Menstrual Needs and Associations with Sexual and Reproductive Risks in Rural Kenyan Females: A Cross-Sectional Behavioral Survey Linked with HIV Prevalence. J Womens Health (Larchmt).

[16] Montgomery P, Hennegan J, Dolan C (2016). Menstruation and the Cycle of Poverty: A Cluster Quasi-Randomised Control Trial of Sanitary Pad and Puberty Education Provision in Uganda. PLoS ONE.

[17] Shah V, Nabwera HM, Sosseh F (2019). A rite of passage: a mixed methodology study about knowledge, perceptions and practices of menstrual hygiene management in rural Gambia. BMC Public Health.

[18] Tembo M (2020). Menstrual Product Choice and Uptake among Young Women in Zimbabwe: A Pilot Study.

[19] UNICEF (2019). Guidance on Menstrual Health and Hygiene.

[20] UNICEF/Zimstats (2019). Zimbabwe 2019 mics suvery findings report.

[21] Tembo M, Simms V, Weiss HA (2024). High uptake of menstrual health information, products and analgesics within an integrated sexual reproductive health service for young people in Zimbabwe. Reprod Health.

[22] Katova L (2022). Population and Housing Census. www.zimbabwe.opendataforafrica.org/anjlptc/2022-population-housing-census-preliminary.

[23] TheWorldBank (2022). Zimbabwe overview. https://www.worldbank.org/en/country/zimbabwe/overview.

[24] Zimbabwe Poverty and Equity Brief (2025). April 2025, in poverty and equity drief washington.

[25] Mangundu M, Roets L, Janse van Rensberg E (2020). Accessibility of healthcare in rural Zimbabwe: The perspective of nurses and healthcare users. Afr J Prim Health Care Fam Med.

[26] Chari A, von Fintel D, Burger R (2025). Inequality in Public Health Spending and Access to Healthcare Services in Zimbabwe: A Cross-Sectional Study. Health Sci Rep.

[27] Ndlovu E, Bhala E (2016). Menstrual hygiene - A salient hazard in rural schools: A case of Masvingo district of Zimbabwe. Jamba.

[28] Phipps AI, Ichikawa L, Bowles EJA (2010). Defining menopausal status in epidemiologic studies: A comparison of multiple approaches and their effects on breast cancer rates. Maturitas.

[29] Mackworth-Young CRS, Wringe A, Clay S (2022). Critical Reflections on Individual Collages as a Research Method With Young Women Living With HIV in Zambia. Emerging Adulthood.

[30] Braun V, Clarke V (2006). Using thematic analysis in psychology. Qual Res Psychol.

[31] Halcomb EJ, Davidson PM (2006). Is verbatim transcription of interview data always necessary?. Appl Nurs Res.

[32] Literat I (2013). “A Pencil for your Thoughts”: Participatory Drawing as a Visual Research Method with Children and Youth. Int J Qual Methods.

[33] Birks M, Chapman Y, Francis K (2008). Memoing in qualitative research:Probing data and processes. J Res Nurs.

[34] Bucher E, Sharkey C, Henderson A (2025). An evaluation of menstrual health apps’ functionality, inclusiveness, and health education information. BMC Womens Health.

[35] Kalampalikis A, Chatziioannou SS, Protopapas A (2022). mHealth and its application in menstrual related issues: a systematic review. Eur J Contracept Reprod Health Care.

[36] Drew S, Buwu N, Gregson CL (2025). Understanding experiences and views of the menopause in Zimbabwe and South Africa: a qualitative study. Climacteric.

[37] Drew S, Khutsoane K, Buwu N (2022). Improving Experiences of the Menopause for Women in Zimbabwe and South Africa: Co-Producing an Information Resource. Soc Sci.

[38] Goffman E (1963). Stigma; Notes on the Management of Spoiled Identity.

[39] Olson MM, Alhelou N, Kavattur PS (2022). The persistent power of stigma: A critical review of policy initiatives to break the menstrual silence and advance menstrual literacy. PLOS Glob Public Health.

[40] Miller TA, Farley M, Reji J (2024). Understanding period poverty and stigma: Highlighting the need for improved public health initiatives and provider awareness. J Am Pharm Assoc (2003).

[41] Islam RM, Rana J, Katha S (2025). Menopause in low and middle-income countries: a scoping review of knowledge, symptoms and management. Climacteric.

[42] Crankshaw TL, Manzini-Matebula N (2025). The continuum of menstrual health through the life course. Lancet Glob Health.

[43] Delanerolle G, Phiri P, Elneil S (2025). Menopause: a global health and wellbeing issue that needs urgent attention. Lancet Glob Health.

[44] Jiao M, Liu X, Ren Y (2021). Comparison of Herbal Medicines Used for Women’s Menstruation Diseases in Different Areas of the World. Front Pharmacol.

[45] Suttor H, Yamayanti KP, Astiti NLEP (2024). Seeking and encountering online information for menstrual health: a qualitative study among adolescent schoolgirls in Gianyar Regency and Denpasar City, Bali, Indonesia. Sexual and Reproductive Health Matters.

[46] Lyzwinski L, Elgendi M, Menon C (2024). Innovative Approaches to Menstruation and Fertility Tracking Using Wearable Reproductive Health Technology: Systematic Review. J Med Internet Res.

