# Supplementary material for: Defined by blood: lived experiences of menstrual health through the lifecourse among women in Zimbabwe – a qualitative study
Source: BMJ Open. 2026 Jul 2;16(7):e113914. doi: 10.1136/bmjopen-2025-113914 (PMC13331150; doi:10.1136/bmjopen-2025-113914)
Supplement: Supplementary file 1 [file bmjopen-2025-113914.reviewer_comments.pdf]

PEER REVIEW HISTORY

BMJ Open publishes all reviews undertaken for accepted manuscripts. Reviewers are asked to complete a checklist review form and are provided with free text boxes to elaborate on their assessment. These free text comments are reproduced below.

ARTICLE DETAILS

Title (Provisional)

Defined by blood: lived experiences of menstrual health through the lifecourse among women in Zimbabwe: A qualitative study

Authors

Tembo, Mandikudza; Weiss, Helen Anne; Nzombe, Portia; Nyamayaro, Chipo Ashley; Hove, Fadzai; Kandiye, Faith R; Dauya, E; Ferrand, Rashida; Mackworth-Young, Constance R.S.

VERSION 1 - REVIEW

**Reviewer**	**1**
**Name**	**Chen, Duan-Rung**
**Affiliation**	**National Taiwan University, Institute of Health Behaviors and Community Sciences**
**Date**	**25-Dec-2025**
**COI**	**None**

As this paper aims to examine generational differences in experiences and perceptions of menstruation, the current age categorization appears overly coarse. It is recommended that menopausal status or age at menopause be explicitly considered. In addition, the second age group spans an excessively wide range and should be further subdivided. A theoretically grounded generational classification, followed by systematic comparative analysis, would better capture intergenerational differences in menstrual perceptions and constitute the paper's primary contribution.

When quoting participants' narratives, it is recommended to annotate each quotation with the participant's age to enhance contextual clarity.

The background information provided for the interviewees is relatively limited. Including additional details on participants' socioeconomic characteristics would strengthen the interpretation of the findings.

The methodological approach employed in this study has inherent limitations. While it allows data collection from a large number of participants simultaneously, it may constrain disclosure of more sensitive or private experiences due to the public nature of the discussion. These limitations should be explicitly acknowledged in the limitations section, along with a discussion of potential strategies to address or mitigate them in future research.

In presenting the results, it is recommended to clearly and systematically compare perspectives across different generations. Such a structured comparison would more effectively highlight both shared and divergent views among generations.

**Reviewer**	**2**
**Name**	**Pike, Meghan**
**Affiliation**	**Dalhousie University**
**Date**	**05-Feb-2026**
**COI**	**None**

It was such a pleasure to review this paper. It is an extremely important topic, not typically studied in developing countries. I loved the 'collage' methodology and would love to try this with my patients. That is a great way to obtain someone's perspective of their own lived experiences. One major strength of this paper, as you pointed out, is the representation of women's experiences from across the lifespan. This is an extremely well written article that reads well. Great work. I look forward to seeing it published and just have some minor comments to improve the scientific merit of the work.

Table 1. Why split age groups this way? This should be justified in text.

For "team discussions" and "group discussions:" what questions were asked?

elaborate on how team discussions contributed to workshop topics. who led these discussions and what were the outcomes?

what is body mapping exercise? not clear in methods.

During analysis, we focused on understanding participants' experiences of MH, managing

18 menstruation, and seeking MH-related healthcare and support. Objectives should explicitly state that these were the outcome measures you were looking for.

how exactly were patients recruited? how could recruitment methods have impacted the results - i.e. if social media used for recruitment, you may miss out on women who dont have a phone

Do you think the fact that there were no local researchers could impact results?

"heavy and/or irregular bleeding due to family planning methods." What do you mean by this?

Giving some background on the health care system earlier on in the manuscript would be helpful - perhaps where you describe the geography. i.e. public or private? how to access physicians?

The study also highlights the subsequent rise in the use of other unregulated and under-researched methods and resources to manage MH-related issues such as MH-focused technology and media platforms. be careful here - do not assume all MH-focused technology and media platforms are under-researched!

VERSION 1 - AUTHOR RESPONSE

	Reviewer Comments	Author Responses
**Reviewer 1**		

**1.**	As this paper aims to examine generational differences in experiences and perceptions of menstruation, the current age categorization appears overly coarse. It is recommended that menopausal status or age at menopause be explicitly considered. In addition, the second age group spans an excessively wide range and should be further subdivided. A theoretically grounded generational classification, followed by systematic comparative analysis, would better capture intergenerational differences in menstrual perceptions and constitute the paper's primary contribution.	Thank you for your comment. This approach to age stratification is noted. Unfortunately, we do not have participants' age at menopause but, in response to your comment, we have further subdivided the second age group to reflect midlife women (36-55 years old) and post-menopausal women (55+ years old). This categorization was based on the post-menopausal cut-off suggested by the *Phipps et al* study. We have also included more information in the manuscript:<br><br>*"The criterion for this study was women across different life stages regarding the menstrual cycle. Participant age categorization was informed by Phipps et al (28) study that suggests postmenopausal cut-off age of 55 years old. We therefore used a categorization of younger (reproductive-age) women aged 18 – 35 years old and older women, including both midlife (perimenopausal) women aged 36 – 55 years old and post-menopausal women aged 55+ years old."*<br><br>**Page 9, Lines 8 - 13**<br>**Page 9, Table 1**
**2.**	When quoting participants' narratives, it is recommended to annotate each quotation with the participant's age to enhance contextual clarity.	We revised the manuscript to include participant ages for all quotes.
**3.**	The background information provided for the interviewees is relatively limited. Including additional details on participants' socioeconomic characteristics would strengthen the interpretation of the findings.	We have included information on the participants' socioeconomic status in the methods section here:<br><br>*"All participants came from low-income households situated in high density areas. Most households in these areas have limited access to WASH facilities, with most accessing water via a public well and some having access to a toilet and washing facilities in the household and other sharing access to a toilet and washing facilities outside the household."*<br><br>**Page 8, Lines 9 - 13**

**4.**	The methodological approach employed in this study has inherent limitations. While it allows data collection from a large number of participants simultaneously, it may constrain disclosure of more sensitive or private experiences due to the public nature of the discussion. These limitations should be explicitly acknowledged in the limitations section, along with a discussion of potential strategies to address or mitigate them in future research.	Thank you for your comment. We agree that the methods chosen had the inherent limitations that the reviewer mentioned. We have elaborated on these, in the strengths and limitations section, as suggested here:<br><br>*"The public and social format of the participatory workshops may have constrained disclosure, especially around particularly sensitive or private experiences or opinions around menstrual health. In future research, this limitation could be mitigated by including follow-up in depth interviews to further explore sensitive experiences in a more private setting."*<br><br>**Page 23, Lines 9 - 13**
**5.**	In presenting the results, it is recommended to clearly and systematically compare perspectives across different generations. Such a structured comparison would more effectively highlight both shared and divergent views among generations.	Thank you for your comment.<br><br>We acknowledge the merits of presenting the results in this way. However, many of the experiences were similar across age-groups, so presentation of results thematically made more sense. Given this rationale, we chose to present the results by grouping them according to themes and subthemes that emerged in through the analysis. We also then highlight the converging and diverging experiences between younger and older women within each theme and elaborate on this in the discussion.
**Reviewer 2**		
**1.**	It was such a pleasure to review this paper. It is an extremely important topic, not typically studied in developing countries. I loved the 'collage' methodology and would love to try this with my patients. That is a great way to obtain someone's perspective of their own lived experiences. One major strength of this paper, as you pointed out, is the representation of women's experiences from across the lifespan. This is an extremely well written article that reads well. Great work. I look forward to seeing it published and just have some minor comments to improve the scientific merit of the work.	Thank you so much for the affirming comments and for reviewing this paper. We really appreciate the kind and positive words.

**2.**	Table 1. Why split age groups this way? This should be justified in text.	Thank you for comment. We have included this justification in the manuscript:<br><br>*"The criterion for this study was women across different life stages regarding the menstrual cycle. Participant age categorization was informed by Phipps et al (28) study that suggests postmenopausal cut-off age of 55 years old. We therefore used a categorization of younger (reproductive-age) women aged 18 – 35 years old and older women, including both midlife (perimenopausal) women aged 36 – 55 years old and post-menopausal women aged 55+ years old."*<br><br>**Page 9, Lines 8 - 13**

**3.**	For "team discussions" and "group discussions:" what questions were asked? Elaborate on how team discussions contributed to workshop topics. who led these discussions and what were the outcomes?	Thank you for your comment. We have included text in the manuscript to make this clearer:<br><br>*"We developed semi-structured topic guides for the workshops, informed by findings from a previous study with young women in the same setting (17, 28) and research team discussions (with HW and CRSMY and led by MT). Each workshop included group member introductions and guided small (two to three participants) and large (all participants together) group discussions. The discussion questions and activities were used to explore participants' perspectives on, and lived experiences of managing menstruation, and seeking healthcare for MH-related issues."*<br><br>**Page 10, Lines 9 - 12**
**4.**	What is body mapping exercise? not clear in methods.	We have revised the manuscript to remove this from the text as we do not include this in the results.

**5.**	"During analysis, we focused on understanding participants' experiences of MH, managing menstruation, and seeking MH-related healthcare and support." Objectives should explicitly state that these were the outcome measures you were looking for.	We have revised the manuscript to reflect this as the aim of the study:<br><br>*"The aim of this study was to understand the menstrual experiences across the lifecourse of women in Zimbabwe, including investigating managing menstruation, MH-related pain and discomfort, and the barriers and facilitators to seeking MH-related healthcare and support."*<br><br>**Page 7, Lines 16 – 18**
**6.**	How exactly were patients recruited? how could recruitment methods have impacted the results - i.e. if social media used for recruitment, you may miss out on women who dont have a phone	Thank you for this question. We have revised the manuscript to make the recruitment process clearer:<br><br>*"We recruited women through clinic and community outreach. Trained female qualitative research assistants (RAs) (PN, CAN, FH, FRK), contacted participants in person or via phone call with information about the study and invited to enrol."*<br><br>**Page 8, Lines 23 -25**

**7.**	Do you think the fact that there were no local researchers could impact results?	Local researchers were involved in the study. We have revised the manuscript text to clarify this:<br><br>*"The research team consisted of nine women who identify themselves as cis-gendered. Five are Zimbabwean and local to the study area"*<br><br>**Page 12, Line 1**
**8.**	"heavy and/or irregular bleeding due to family planning methods." What do you mean by this?	We have included examples of family-planning methods that are known to cause heavy and/or irregular bleeding in the manuscript here:<br><br>*"heavy and/or irregular bleeding due to family planning methods such as the hormonal contraceptive implant or the copper intrauterine device"*<br><br>**Page 16, Lines 3 - 4**

**9.**	Giving some background on the health care system earlier on in the manuscript would be helpful - perhaps where you describe the geography. i.e. public or private? how to access physicians?	Thank you for this comment. We have revised the manuscript as advised here:<br><br>*"Most Zimbabweans cannot afford private healthcare and public healthcare access is limited by systemic challenges, including under-resourced facilities, geographic disparities in service availability and associated high transport costs, unaffordable user fees, and limited skilled healthcare personnel and essential medical supplies, particularly in maternal and reproductive health (25, 26)."*<br><br>**Page 8, Lines 9 – 13**
**10.**	The study also highlights the subsequent rise in the use of other unregulated and under-researched methods and resources to manage MH-related issues such as MH-focused technology and media platforms. be careful here - do not assume all MH-focused technology and media platforms are under-researched!	Thank you for this comment. We acknowledge that not all MH-focused technology is under-researched. We have revised this in the manuscript to highlight specific research gaps:<br><br>*"The study also highlights the subsequent rise in the use of other largely unregulated and under-researched methods and resources to manage MH-related issues, particularly MH-focused technology and media platforms. These remain comparatively insufficiently studied, with notable gaps in evidence regarding their effectiveness, inclusivity, safety, and overall impact on users (35, 36)"*

		**Page 20, Lines 22 -26**

VERSION 2 - REVIEW

Reviewer 1

Name Chen, Duan-Rung

Affiliation National Taiwan University, Institute of Health Behaviors and Community Sciences

Date 24-May-2026

COI

The revised manuscript addresses an important and under-researched public health issue. However, I remain concerned that the paper's stated life-course framing is insufficiently operationalized in the analysis. Although the manuscript includes women from different age groups and now provides additional age information, the results remain largely organized around broad thematic categories rather than a systematic examination of life-course processes, life-stage transitions, cohort differences, or cumulative experiences across women's lives.

The distinction between including participants of different ages and adopting a life course perspective needs to be made more explicit. A genuine life course analysis would require clearer attention to how menstrual meanings, stigma, bodily experiences, health literacy, care-seeking behavior, and access to information change from menarche through reproductive adulthood, perimenopause, menopause, and later life. At present, the paper identifies some age-related differences, such as younger women's greater use of digital resources and older women's limited knowledge of menopause, but these observations are not developed into a coherent life course argument.

As a result, the revision improves descriptive clarity but does not substantially strengthen the manuscript's conceptual contribution. The findings now read as a relatively general qualitative account of menstrual stigma, secrecy, pain normalization, limited product choice, constrained healthcare access, traditional remedies, and digital information seeking. These are important issues, but they are not sufficiently novel unless they are analytically connected to life course mechanisms and intergenerational differences. In this sense, the revised results have become more ordinary and less innovative than the manuscript's title and framing would lead readers to expect.

I therefore recommend that the authors either substantially strengthen the life course analysis or moderate the paper's claims. If the authors wish to retain the life-course framing,

the results and discussion should be reorganized to more systematically compare younger, midlife/perimenopausal, and postmenopausal women, and to show how early menstrual experiences shape later perceptions, silence, care-seeking, and embodied identity. Alternatively, the authors should present the study more modestly as a qualitative exploration of menstrual health experiences among adult women of different ages in Zimbabwe, rather than as a fully developed life-course analysis.

Overall, while the manuscript is valuable, its current revision does not yet deliver the conceptual originality promised by the life course framing. A more explicit and theoretically grounded life-stage comparison would substantially improve the paper's analytical depth, innovation, and contribution to menstrual health research.

VERSION 2 - AUTHOR RESPONSE

<table>
<tr><td colspan="3">Reviewer 1</td></tr>
<tr><td>1.</td><td>The revised manuscript addresses an important and under-researched public health issue. However, I remain concerned that the paper's stated life-course framing is insufficiently operationalized in the analysis. Although the manuscript includes women from different age groups and now provides additional age information, the results remain largely organized around broad thematic categories rather than a systematic examination of life-course processes, life-stage transitions, cohort differences, or cumulative experiences across women's lives.

The distinction between including participants of different ages and adopting a life course perspective needs to be made more explicit. A genuine life course analysis would require clearer attention to how menstrual meanings, stigma, bodily experiences, health literacy, care-seeking behavior, and access to information change from menarche through reproductive adulthood, perimenopause, menopause, and later life. At present, the paper identifies some age-related differences, such as younger women's greater use of digital resources and older women's limited knowledge of menopause, but these observations are not developed into a coherent life course argument.

As a result, the revision improves descriptive clarity but does not substantially strengthen the manuscript's conceptual contribution. The findings now read as a relatively general qualitative account of menstrual stigma, secrecy, pain normalization, limited product choice, constrained healthcare access, traditional remedies, and digital information seeking. These are important issues, but they are not sufficiently novel unless they are analytically connected to life course mechanisms and intergenerational differences. In this sense, the revised results have become more ordinary and less</td><td>Thank you for your comprehensive and thoughtful feedback.

Overall, we found the feedback very helpful and have made the following changes:

We have expanded the lifecourse analysis throughout the results section through adding and expanding analysis that more explicitly discusses experiences alongside life stage. These include the similarities and differences between experiences at different ages and how menstrual experiences earlier in life impact menstrual experiences and perceptions later in life. Please see the changes below:

"Almost all participants, regardless of age, felt menstruation marked the beginning of "womanhood" as it signified that one's body was biologically ready to conceive a child."

Page 12

"Older participants added that these negative experiences at menarche continued to negatively inform their perceptions and experiences around menstruation and MH-related issues throughout their lives. Some participants expressed feeling relieved of "burden" once in menopause"

"Periods have always felt like a burden. It is continuous work and pain. I was</td></tr>
</table>

	innovative than the manuscript’s title and framing would lead readers to expect.<br><br>I therefore recommend that the authors either substantially strengthen the life course analysis or moderate the paper’s claims. If the authors wish to retain the life-course framing, the results and discussion should be reorganized to more systematically compare younger, midlife/perimenopausal, and postmenopausal women, and to show how early menstrual experiences shape later perceptions, silence, care-seeking, and embodied identity. Alternatively, the authors should present the study more modestly as a qualitative exploration of menstrual health experiences among adult women of different ages in Zimbabwe, rather than as a fully developed life-course analysis.<br><br>Overall, while the manuscript is valuable, its current revision does not yet deliver the conceptual originality promised by the life course framing. A more explicit and theoretically grounded life-stage comparison would substantially improve the paper’s analytical depth, innovation, and contribution to menstrual health research.<br>.	**actually happy when they stopped.” – mid 50s Harare**<br><br>**Page 14**<br><br>“In describing their menarcheal experiences, many participants reported using tissue or cloth for their first period as they were unprepared and afraid to seek advice on what do and what to use (see Figure 5). Feeling unprepared and having a lack of options for advice was universal among older women, while some younger women noted being able to ask a friend for assistance.”<br><br>**Page 17**<br><br>We have also moderated the paper’s claims as follows:<br><br>“We add a temporal lens to the model, elaborating on proximal and distal antecedants of menstrual experiences through to the impacts of this at different ages and exploring the similarities and differences of lived MH experiences across generations of women.”<br><br>**Page 21**<br><br>While we have expanded the life course analysis in the results, we have decided not to re-organise the results by age-group. Since menstrual experiences were interestingly more similar across ages, we feel the thematic structure of the results section adds more value, and the lifecourse analysis adds where experiences were similar or different across ages, and where experiences early in life impacted those later in life, which are included throughout the results and expanded upon in the discussion.<br>We hope the changes made and explanations given adequately address the concerns raised by the reviewer.
